# Analyzing cachectic phenotypes in the muscle and fat body of *Drosophila* larvae

**DOI:** 10.1016/j.xpro.2022.101230

**Published:** 2022-03-09

**Authors:** Callum Dark, Shane Cheung, Louise Y. Cheng

**Affiliations:** 1 Peter MacCallum Cancer Centre, Melbourne, VIC 3000, Australia; 2 Sir Peter MacCallum Department of Oncology, The University of Melbourne, Melbourne, VIC 3010, Australia; 3 Biological Optical Microscopy Platform, Faculty of Medicine, Dentistry & Health Sciences, The University of Melbourne, Melbourne, VIC 3010, Australia; 4 Department of Anatomy and Physiology, The University of Melbourne, Melbourne, VIC 3010, Australia

**Keywords:** Developmental biology, Metabolism, Model Organisms

## Abstract

*Drosophila* has become a popular model for examining the metabolic wasting syndrome, cachexia, characterized by degradation of muscles and fat. Here we present a protocol for quick and consistent scoring of muscle detachment, fat body lipid droplet size, and extracellular matrix (ECM) quantifications in *Drosophila* larvae. We detail the procedures for dissecting, staining, and imaging third instar *Drosophila* larval muscle fillets and fat body, and how to utilize FIJI macros for robust quantification of cachectic phenotypes in these dissected tissues.

For complete details on the use and execution of this protocol, please refer to Lodge et al. (2021).

## Highlights

• Detailed description of larval muscle preparation for muscle detachment analysis
• Detailed description of fat body dissection
• Quantification of fat body lipid droplet size and ECM disruption using FIJI macros

## Graphical abstract

**Figure:**
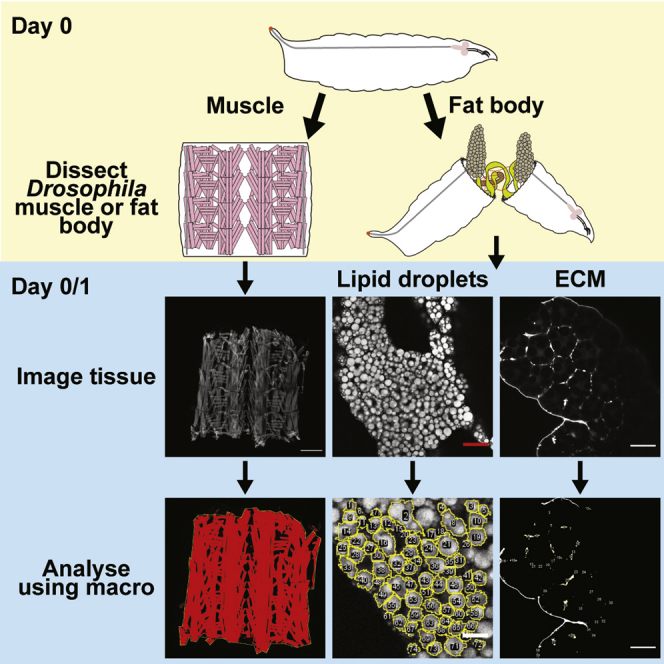

## Before you begin

*Drosophila* larvae have been increasingly used to study the wasting phenotypes seen in cachexia (Ding et al., 2021; Figueroa-Clarevega and Bilder, 2015; Kwon et al., 2015; Newton et al., 2020; Santabárbara-Ruiz and Léopold, 2021; Song et al., 2019). In our recent study, we have shown that tumor bearing animals exhibit several wasting phenotypes including muscle detachment, as well as fat body lipid droplet accumulation and extracellular matrix (ECM) mislocalization (Lodge et al., 2021). As utilization of *Drosophila* larvae to study cachexia has become increasingly popular, and the field is rapidly expanding, it is important to have standardized and quantitative methods that enable phenotypes between studies or in different genetic models to be easily compared. Here we present a collection of protocols for systematic quantification of cachexia phenotypes in the *Drosophila* muscle and the fat body, using semi-automated FIJI macros (Schindelin et al., 2012). While these protocols are devised to primarily characterize tumor induced cachexia phenotypes, they can also be used in other disease settings such as muscular dystrophy (muscle detachment) or obesity (lipid droplet accumulation). Furthermore, these quantitative methods can also be adapted for drug or genetic screens, to ensure easy and rapid comparison between experimental settings.

### Prepare solutions

**Timing: 10 min per solution**

There are a number of solutions that will be used in this protocol that should be prepared in advance.

1. 1× PBS can be prepared in advance by diluting 10× PBS in dH_2_O. 1× PBS can be stored at room temperature (RT; 20°C–25°C) for up to six months.
  a. For the cold 1× PBS that is used later in the ‘step-by-step method details’ section (steps 1, 7, 8, 9, 41, and 42), we recommended storing around 50 mL of 1× PBS at 4°C. This can be stored for up to six months.
2. ∼4% Formaldehyde in PBS is recommended to be prepared fresh by making a 1:10 dilution of 36.5%–38% Formaldehyde solution in 1× PBS. However, once prepared it can be used for up to two weeks while stored at room temperature (RT; 20°C–25°C).
3. 0.2% and 0.3% Triton X in 1× PBS (PBST) can be prepared in advance and stored at room temperature (RT; 20°C–25°C) for up to four weeks.
  a. It is recommended to first make a 10% Triton X in 1× PBS solution, and then dilute from there. The 10% Triton X solution can be stored at room temperature (RT; 20°C–25°C) for up to six months.
4. 50% and 80% glycerol in 1× PBS can be prepared in advance and stored at room temperature (RT; 20°C–25°C) for up to eight weeks.

## Key resources table

**Table:** 

REAGENT or RESOURCE	SOURCE	IDENTIFIER
**Antibodies**		

Alexa Fluor™ 647 Phalloidin (Concentration: 1:200)	Thermo Fisher Scientific	Cat#A22287
DAPI 405 (Concentration 1:100)	Thermo Fisher Scientific	Cat#D1306
HCS LipidTOX™ Deep Red Neutral Lipid Stain (Concentration 1:1000)	Thermo Fisher Scientific	Cat#H34477

**Chemicals, peptides, and recombinant proteins**		

10× PBS	Thermo Fisher Scientific	Cat#70011044
Triton X-100	Sigma-Aldrich	Cat#93443
36.5%–38% Formaldehyde Solution	Sigma-Aldrich	Cat#47608
100% Glycerol	Sigma Aldrich	Cat#G5516

**Experimental models: Organisms/strains**		

*D. melanogaster*, third instar larvae: Expresses mCherry-tagged rhea protein: y[1] w[∗]; Mi{PT-mCh.0}rhea[MI00296-mCh.0] lncRNA:CR43910[MI00296-mCh.0-X]/TM6B, Tb[1]	Bloomington Drosophila Stock Center	BDSC: 39648; FlyBase: FBst0039648
*D. melanogaster*, third instar larvae: Expresses GFP-tagged Viking (collagen-IV) protein: w[∗]; P{w[+mC]=PTT-un1}G00454	Kyoto Stock Center (DGRC)	DGRC: 110692; FlyBase: FBti0153267
*D. melanogaster*, third instar larvae: Expresses YFP-tagged trol protein: PBac{681.P.FSVS-1}trol[CPTI002049] w[1118]	Kyoto Stock Center (DGRC)	DGRC: 115262; FlyBase: FBst0325254
*D. melanogaster*, third instar larvae: Expresses GFP-tagged Nidogen protein: PBac{fTRG00638.sfGFP-TVPTBF}VK00033	Vienna Drosophila Resource Center	VDRC: 318629; FlyBase: FBst0491653

**Software and algorithms**		

FIJI, Version 1.53 or later	National Institutes of Health, USA, (Schindelin et al., 2012)	https://imagej.net/software/fiji/
FV31S-SW viewer software	Olympus	Available upon quote request from Olympus
GraphPad Prism 9.0.2	GraphPad	https://www.graphpad.com/
Oir to tiff macro	This paper	N/A
Fillet muscle detachment analysis	This paper	N/A
Lipid droplet quantification analysis_singlechannel	This paper	N/A
Lipid droplet quantification analysis_multichannel	This paper	N/A

**Other**		

Corning® 40 μm Cell Strainer	Corning	Cat#431750
6-well culture plate	Thermo Fisher Scientific	Cat#140675
Petri dishes, polystyrene, 60 mm × 15 mm	Sigma-Aldrich	Cat#P5481
100 mL Conical flask	Thermo Fisher Scientific	Cat#2121624
Tile cavity 6 place porcelain black spotting plate	Industrial Equipment & Control PTY. LTD.	Cat#LW5519-02
Thermometer	Thermo Fisher Scientific	Cat#44-442-0
Dissecting Pad, Clear	Living Systems Instrumentation	Cat#DD-90-S
Taklon Paint brush, Size 6	Australian Entomological Supplies PTY LTD	Cat#EBT6
High Precision Straight Tapered Ultra Fine Point Tweezers/Forceps	Thermo Fisher Scientific	Cat#12-000-122
Micro-Headless Pins A1 - 0.142 mm × 10 mm (Dissecting pins)	Australian Entomological Supplies PTY LTD	Cat#E183
Mini Spring Scissors (Dissecting scissors)	Australian Entomological Supplies PTY LTD	Cat#E145A
Series 2 Adhesive Microscope Slides (Glass)	Trajan	Cat#472042491
Series 1 coverslip (24 mm × 24 mm, Glass)	Trajan	Cat#471112424
Rimmel 60 Seconds Nail Polish Clear	Chemist Warehouse (or any similar supplier, depending on country)	Cat#2651592
Scotch® Magic™ Tape	Scotch™ Brand	N/A
Scalpel Blade	Thermo Fisher Scientific	Cat#21062-SM
RNase-free Microfuge Tubes (1.5 mL)	Thermo Fisher Scientific	Cat#AM12400
Olympus FLUOVIEW FV3000 confocal laser scanning microscope	Olympus	Available upon quote request from Olympus
UPLFLN10X2 air objective	Olympus	Cat#N2249200
UPLXAPO40X air objective	Olympus	Cat#N5702100

## Step-by-step method details

### Heat killing of *Drosophila* larvae

**Timing: 3 min per larva**

Here we describe a method for heat killing third instar larvae before muscle fillet dissection, which improves the quality of muscle fillets, preserves muscle integrity and reduces muscle detachment artifacts.

***Note:*** Heat killing using high temperatures prevents muscle contractions during death. Additionally, heat killed larvae will not move when one attempts to pin down the larvae, making the process easier. This procedure can be used for other muscle preparations, such as dissections followed by immunostaining.

1. Transfer larvae from vial to a 6-place porcelain spotting plate with cold 1× PBS (Figure 1A).

**Figure 1:**
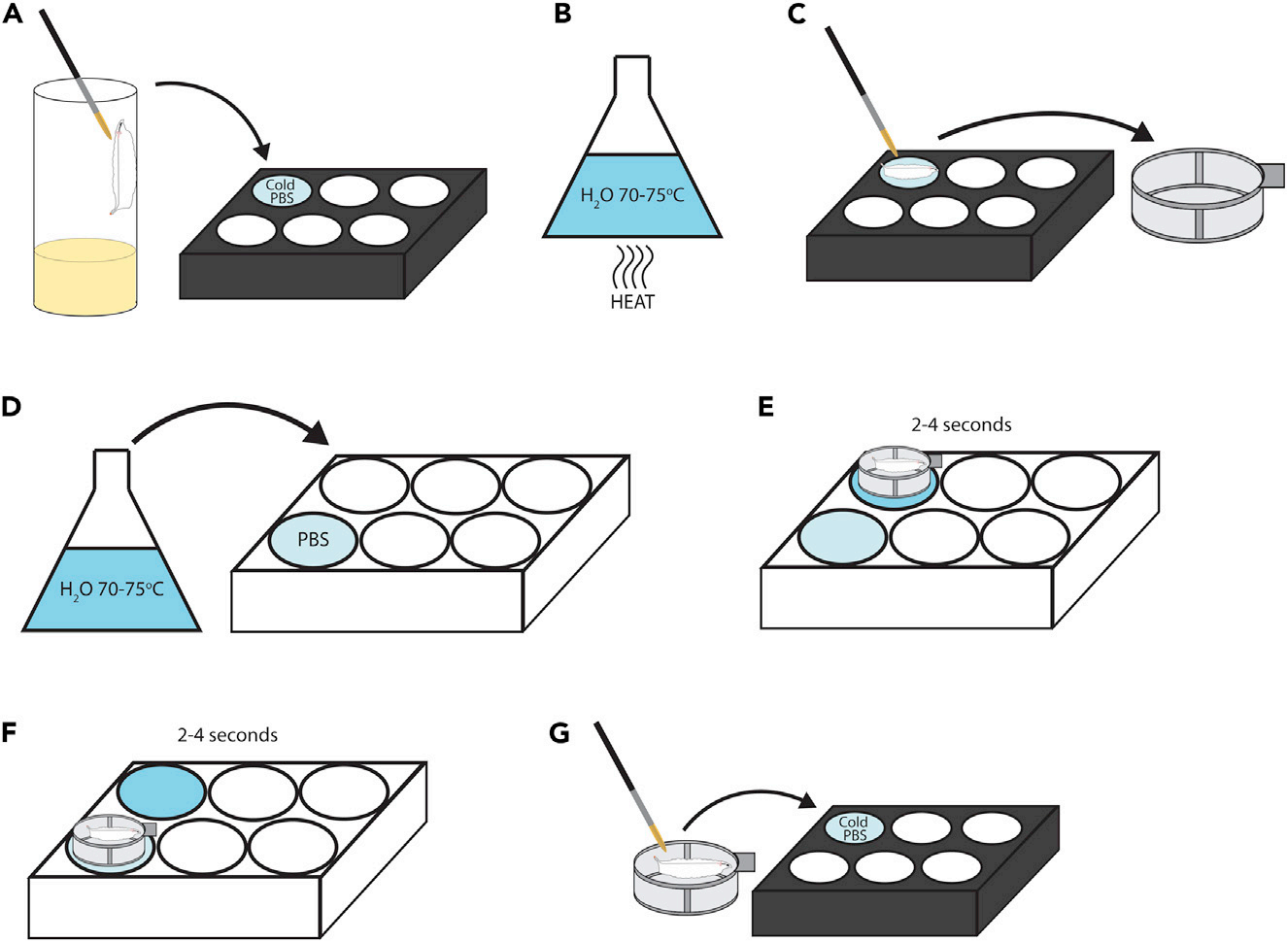
Diagram sequence for heat killing *Drosophila* larvae (A–G) Brief illustrated instructions for the heat killing procedure. Detailed instructions can be found in the main text.
2. Pour some 1× PBS into one of the wells of the 6-well culture plate

***Alternatives:*** Petri dishes can be used instead of the 6-well culture plate.

3. Heat up water in a conical flask using a microwave until boiling or just before boiling (Figure 1B).
4. Transfer larvae from the 6-place porcelain spotting plate to a 40 μm cell strainer using a paint brush (Figure 1C).
5. Measure the temperature of the water until it is around 70°C–75°C, then pour it into a well of the culture plate or petri dish (Figure 1D). The water will cool very quickly once it is poured, so do not wait too long before the next step.

**CRITICAL:** If the water temperature is lower than 70°C, this process is not effective.

6. Dip the cell strainer containing the larvae into the water for 2–4 s (Figure 1E), then remove from the water and dip into the PBS for 2–4 s to cool (Figure 1F).
7. Transfer larvae out of the cell strainer back to a 6-place porcelain spotting plate with cold 1× PBS (Figure 1G).

### Dissection of larval muscle fillets

**Timing: 3 min per larva**

Here we describe the dissection of a muscle fillet from third instar larvae.

***Note:*** The size of the pins can really make a difference when dissecting larval fillets. We recommend using pins with a diameter of 0.142 mm or thinner, as they allow for minimal disruption to the muscle, and more precise placement of the pins. Keep in mind that the thinner pins are more likely to bend at the tip, making them unusable and thus require more delicate use.

**Figure 2:**
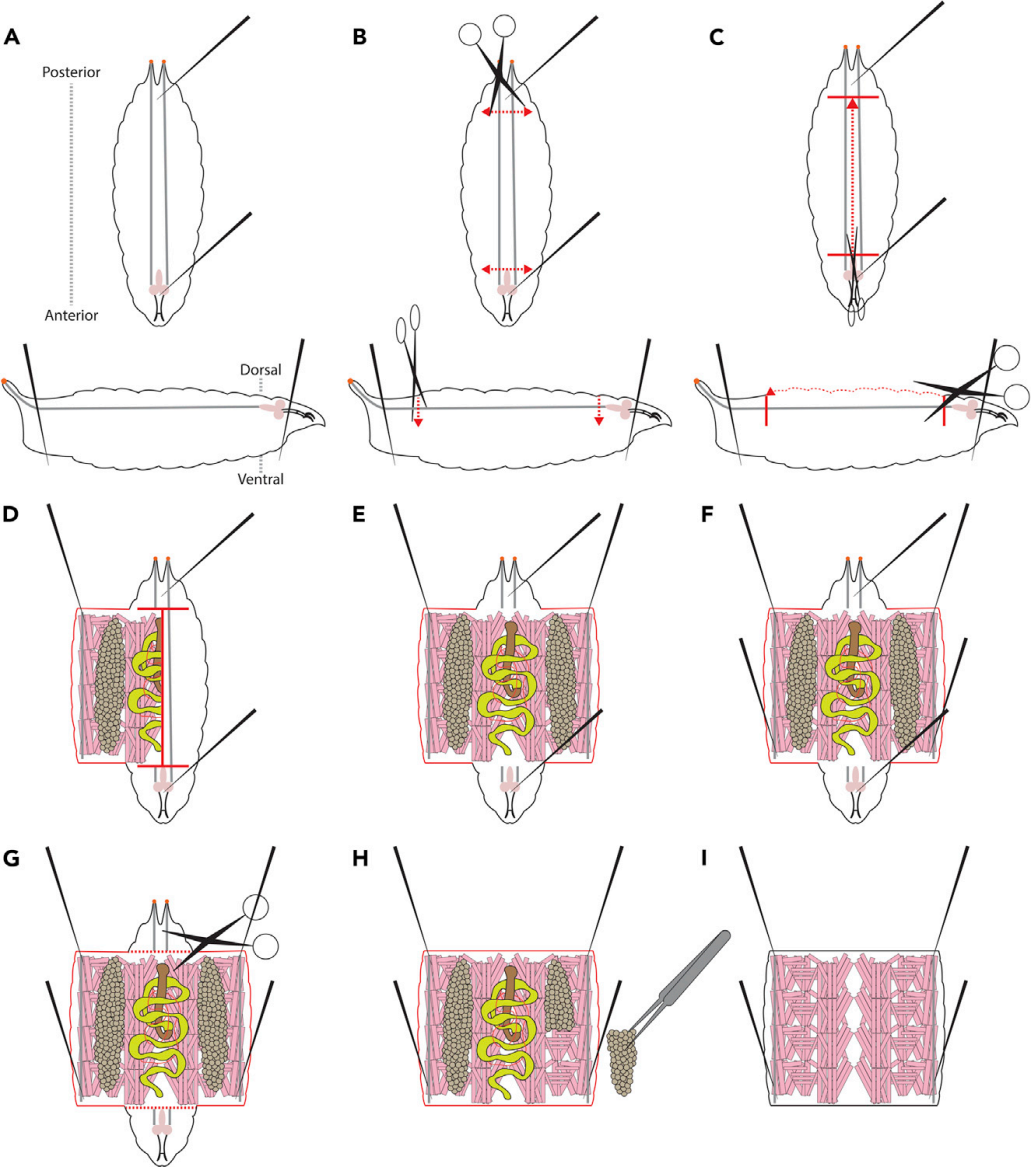
Diagram sequence for dissecting *Drosophila* larval muscle fillets (A–I) Brief illustrated instructions for the larval muscle fillet dissection procedure. Red dotted lines indicate active cutting lines. Red solid lines indicated previously cut lines. Detailed instructions can be found in the main text.
11. Hold the anterior end of the larva, and stretch it out to create light tension, then pin down the anterior end.
12. Using the dissection scissors, make an incision below the pin at the posterior end, cutting through half of the larva, as shown in Figure 2B. Make a similar incision above the pin at the anterior end.
13. Orientating the scissors perpendicular to the incisions made in step 12, cut through the cuticle of the larva at the midpoint of the width of the larva (between the trachea), all the way from the incision made at the anterior end to the posterior end (Figure 2C).
14. Use forceps to pick up and pull back the fillet, until it opens up like a book, and place a pin in the corner of the fillet (Figure 2D). Do the same on the other side of the fillet (Figure 2E).

***Note:*** Which of the four corner pins you pin first is not important, but you should fully pin down either the posterior or anterior side’s pins first (Figure 2E), before doing the next two (Figure 2F).

8. Using a small paintbrush, transfer a single larva from the 6-place porcelain spotting plate containing cold PBS (from step 7) to the dissecting pad (See the key resources table for more information).
9. Pipette 40–50 μL of fresh 1× cold PBS on top of the larva so that it is fully covered.
10. Using forceps and dissecting pins, pin down the posterior end of the larva first so that it is orientated as Figure 2A, placing the pin on the dorsal side of the larva, between the two tracheal branches.

**CRITICAL:** Try to create light tension in the fillet so that it is fully stretched but be careful not to apply too much pressure or you may tear the fillet.

15. Repeat step 14 for the opposite end of the fillet, until it is pinned out as shown (Figure 2F).

***Note:*** The speed at which you dissect may determine how many larvae you do before starting the fixation process. Having a different timer for each larva can become cumbersome, so it is recommended to do multiple larvae and then fix them as a group, perhaps sorting them by genotype. Doing too many before fixing is also not recommended, as the first few larvae can begin to deteriorate, especially if they have been heat killed.

**CRITICAL:** For phalloidin stained muscle fillets, we recommend not leaving the first dissected larvae unfixed for more than 20 min.

***Note:*** To begin with we recommend only doing three larvae per group before fixation. However, it is possible to do up to eight or ten within the 20 min time window.

### Fixation of dissected larval muscle fillets

**Timing: 1 h per larva/group of larvae**

Here we will fix the muscle fillets that we have dissected above. Freshly prepared 4% formaldehyde is recommended.

16. Remove the PBS from the larvae, and add 40–50 μL of 4% Formaldehyde. Fix for 20 min at room temperature (RT) (If using this protocol for antibody stains other than phalloidin, please refer to the manufacturer’s protocol for the best fixation conditions).

***Note:*** While fixing the first group of larvae, you may commence dissection of the next group of larvae to save time.

17. Remove the 4% Formaldehyde, and wash in 40–50 μL of 0.3% PBST for 10 min at RT (repeat 2 times).
  a. During the second wash, remove the pins from either end of the larva, leaving only the 4 pinning down the fillet.
    i. Using the dissecting scissors, remove the head and tail of the fillet, as shown in Figure 2G.
    ii. Remove the internal organs and tissues, including the gut, fat body, central nervous system, and discs (Figure 2H).
      **CRITICAL:** The removal of internal organs is important, as it allows full exposure of muscles to the phalloidin/PBST solution in step 19.
    iii. Try to leave the trachea in this cleaning process, as trachea will help to guide the mounting of the fillet in step 25.
      ***Note:*** Do not worry about removing all the axons that were connected to the brain, as these will not affect the detachment score, and are too difficult to remove.
      **CRITICAL:** Be careful not to touch the muscle with the forceps in this process. The fillet should look like Figure 2I.
  b. During the final wash, remove the final four pins from the fillet, letting it float freely in the PBST.
18. Transfer the fillet to a 6-place porcelain spotting plate containing 0.3% PBST by picking it up on one corner of the fillet, without touching the muscles.

### Staining of fixed larval muscle fillets

**Timing: Either 1.5h, or overnight, as required**

To visualize the muscle fibers attached to the cuticle, we will stain for F-actin using phalloidin diluted in PBST.

19. Remove the PBST and replace with phalloidin diluted in 0.3% PBST.
  a. Make sure there is enough solution to fully cover all the fillets, we find 200 μL per well in the spotting plate is usually enough.
  b. Use any fluorescently-labeled phalloidin that you prefer, and follow the recommended concentrations provided by the manufacturer.
  c. We use a concentration of 1:200 for our phalloidin staining.

***Note:*** We use 6-place porcelain spotting plates for the staining process as we have found it allows even staining of the muscle fillets without agitation.

***Alternatives:*** You can perform the phalloidin staining in a 1.5 ml microcentrifuge tube on a shaker, just be careful not to touch the muscles while transferring the fillets in and out of the microcentrifuge tubes.

20. Stain the fillets in the dark, for either 1 h at RT, or for up to 24 h at 4°C. The phalloidin stain is very robust, so either procedure works.
21. Once the staining is complete, remove the phalloidin solution, and wash in 0.3% PBST three times (10 min each time) at RT.
22. Remove the PBST, and replace with 50% glycerol to fully cover the fillets.

**Pause Point:** These fillets can be stored at 4°C in 50% glycerol for up to a week before mounting. If the fillets were stored at 4°C, store the 6-place porcelain spotting plate in a moist chamber to prevent drying out of the samples (a moist paper towel in a dark box works well).

### Mounting of stained larval muscle fillets

**Timing: 15 min**

Here we will mount the stained muscle fillets ready for imaging.

***Note:*** For quicker imaging, you can mount up to 6 fillets under 1 coverslip (18 mm × 18 mm).

23. Once ready to mount, prepare a glass slide by pipetting 40–50 μL of 80% glycerol onto the slide.
24. Transfer your muscle fillet(s) onto the slide, make sure they are submerged in the glycerol.
25. To ensure they are orientated muscle side up (cuticle side down), find the trachea you have left on the fillet, they should face up. After orientating the fillets, you can then remove the trachea, as well as any residual organs that were left on the muscle fillets.

***Note:*** See “troubleshooting: problem 1”.

26. Line up the fillets so that their anterior/posterior axis runs parallel to the length of the slide, this will help with imaging in the “imaging of larval muscle fillets” section.
27. Adjust the glycerol level on the slide as needed, adding more if the fillets need extra coverage, or removing some if there is too much and it will spill out the edge of the coverslip.
28. Carefully lower a coverslip onto the slide, taking care to avoid creating air bubbles on the fillet. One tip is to make sure the glycerol has made contact at the side of the coverslip first, then lower it down gently over the fillets with a pair of forceps.

***Note:*** Trapped air bubbles can affect the detachment score, so if many air bubbles are trapped between the coverslip and the fillet, you should remount the fillet.

29. Seal the edges of the coverslip using nail varnish.

### Imaging of larval muscle fillets

**Timing: Varies based on confocal**

Here we describe the settings required for imaging the muscle fillets on an Olympus FV3000, however, any confocal microscope is sufficient. Please note that the process can slightly differ depending on the microscope used.

***Note:*** We use an Olympus FV3000 confocal microscope and a 10×/0.3 NA UPlanFL air objective. This low magnification allowed us to image the entire fillet with a low number of images for tiling at multiple positions which are then stitched to create a single image for analysis. The range of the Z-stack was set using a First/ Last mode and adjusted to encompass the full thickness of the fillet with five slices. While the bulk of muscle fluorescence is relatively flat due to the compression from the coverslip, acquiring a z-stack allows us to ensure muscle staining at different focal planes is captured for the most accurate muscle/cuticle analysis.

### Settings required for acquisition

In order to process acquired files with the macro, the following acquisition settings/parameters are used:

30. Set the range of the Z-stack using a First/ Last mode. Five slices are used to create the Maximum Intensity Projection. First, focus the objective on the middle and brightest plane of the fillet, and then adjust the focus above and below this plane until saturation disappears almost entirely from the image in either direction.
  a. Set the gain so that it picks up all the pixels at the top and bottom of the acquisition stack. As there is a significant difference in the fluorescent intensity at the center of the stack compared to the upper and lower slices, it is likely that pixels in the center of the stack will be over saturated. As the images are used to create a mask and we are not making an intensity measurement, this does not influence the muscle area calculated by the macro.
    i. A 10× objective is recommended for acquisition as this provides a large field of view and reduces the number of tiles required to image the whole fillet, while providing sufficient resolution to capture the finer details of the muscle structure.
    ii. The use of a tiling program within the confocal software is recommended, as it is unlikely that the whole fillet will fit in a single field of view. Stitching the images is required before applying the macro. This can be done either within the confocal software (such as FV31S-SW on the Olympus FV3000) or a program such as FIJI (Plugins>Stitching>Grid/Collection stitching)(Preibisch et al., 2009).
    iii. An image size of 512 × 512 pixels provides sufficient resolution for this analysis, while also allowing for relatively fast acquisition and small file size.
    iv. Kalman averaging is not required however, it is a potential option depending on the noise levels on the microscope of choice.

### Analysis of imaged larval muscle fillets

**Timing: 2 min per fillet**

Here we describe a FIJI macro that analyzes the muscle detachment score of dissected muscle fillets stained with phalloidin.

**Figure 3:**
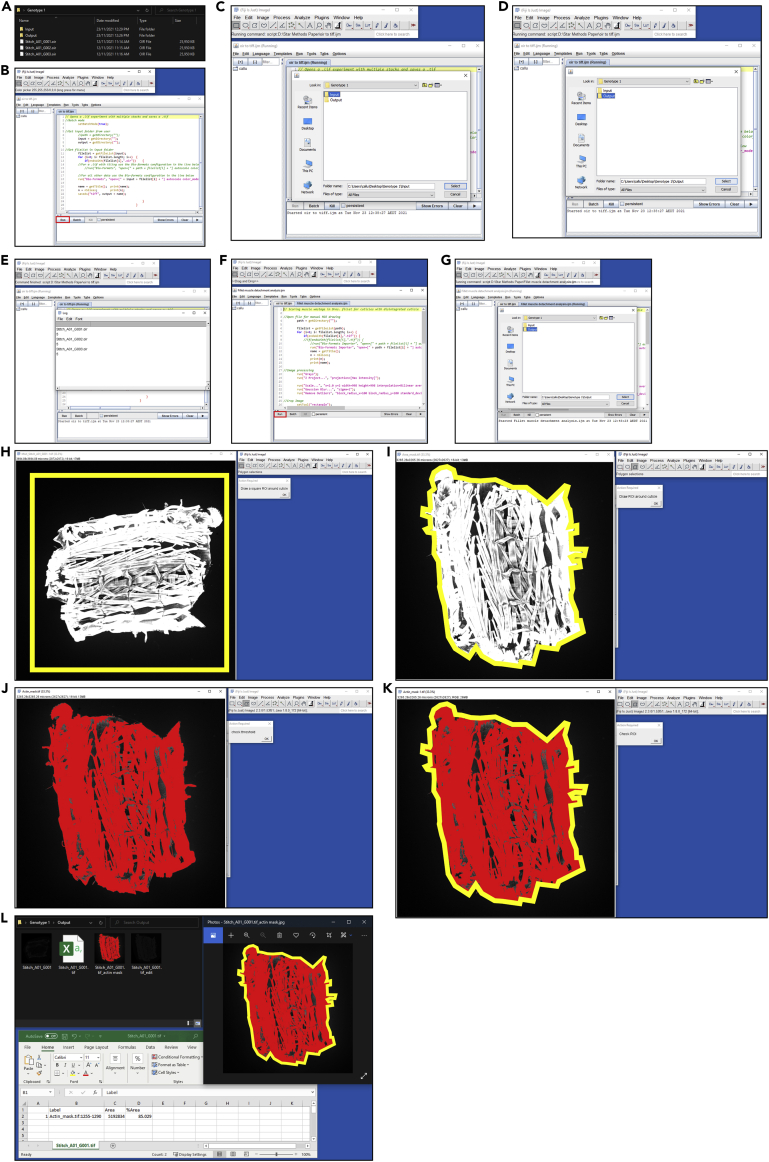
Walkthrough of the muscle fillet macro (A–L) Brief illustrated instructions for the muscle detachment FIJI macro. Detailed instructions can be found in the main text.
33. Import the “oir to tiff” macro into FIJI, and hit ‘Run’ (Figure 3B). Macros can be imported by dragging and dropping the macro file into FIJI. The “oir to tiff” macro is included as an uploaded file associated with this protocol (Data S1).
34. Select the ‘Input’ folder for your first group (Figure 3C), and the ‘Output’ folder for where the macro will deposit the converted files (Figure 3D). It will run through the images in the ‘Input’ folder, and output them as converted tiff files in the ‘Output’ folder (Figure 3E).
35. Once the command has finished, Import the “Fillet muscle detachment analysis” macro into FIJI, and hit ‘Run’ (Figure 3F). The “Fillet muscle detachment analysis” macro is included as an uploaded file associated with this protocol (Data S2).
36. Select the ‘Output’ folder from where you sent the converted files from step 34 (Figure 3G).
37. The command will begin, and will show a number of images of the first fillet. Using the top-most image, follow the commands of the macro, starting with drawing a square around the fillet (Figure 3H). You can create a perfect square by holding Shift on Windows or Mac. While this isn’t a necessary step, it does make for a more consistent image if you want to display the binary masks later on.

***Note:*** If you click ‘OK’ before drawing either the ‘square ROI’ or the ‘fillet ROI’, the macro will encounter an error and will need to be restarted.

**Figure 4:**
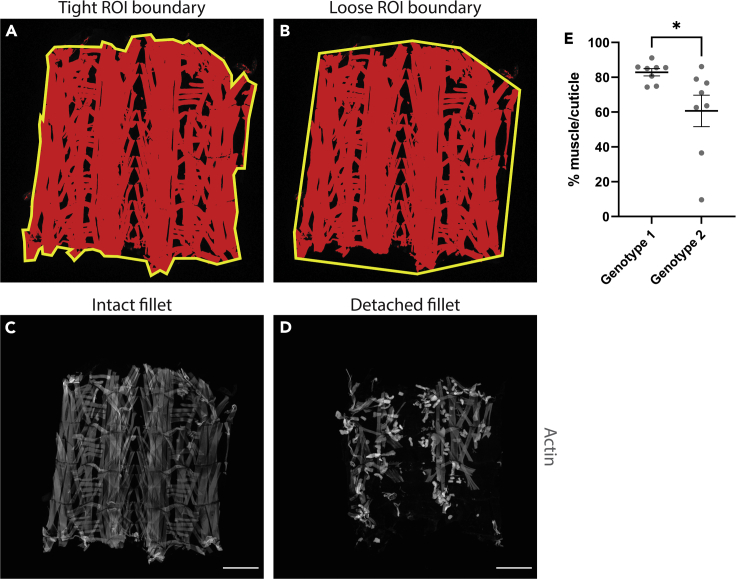
Examples of outputs from the FIJI muscle fillet macro (A and B) Representations of different ROI boundaries. (C) Example of an intact fillet. Scale bar = 500 μm. (D) Example of a detached fillet. Scale bar = 500 μm. (E) Quantification of fillets from C & D. Error bars = ± SEM, ∗ = *p* < 0.05.

31. Separate stitched images into folders according to the experimental conditions used, for example, genotype, drug treatment, etc.
32. Within the respective folders, create an ‘Input’ and an ‘Output’ folder (Figure 3A). Move the images into the ‘Input’ folder for each respective group.

38. Draw an ROI around the fillet (Figure 3I).
  **CRITICAL:** The most important thing is to be consistent between fillets as to the degree of which you stick to the fluorescent boundary, some examples are given in Figures 4A and 4B.
  a. The macro will ask you to confirm the threshold (Figure 3J), and the ROI (Figure 3K). Check they are as expected, and click ‘OK’ in both instances to proceed.
    ***Note:*** See “troubleshooting: problem 2”

39. Proceed through each fillet in the given folder. Each fillet will give a .csv output that represents the percentage of area that the fluorescent pixels occupy per ‘fillet ROI’. An example of the outputs from one fillet is given in Figure 3L. On average, a 5 days post fertilization (dpf) wildtype fillet, such as a w^1118^, will have a percentage muscle value of around 80%–90%, given that gaps between muscles naturally occur in the fillet. The macro calculates the percentage muscle value using the “Area” and “Area fraction” options in “Set Measurements”. Some examples are given in Figures 4C and 4D.
40. Repeat steps 33–39 for the other experimental groups.

### Dissection and fixation of larval fat body

**Timing: 1 h 15 min**

**CRITICAL:** In contrast to muscle, larval fat body can be incredibly delicate. Therefore, we recommend a longer fixation step (40 min) for fat body preparation, and any dissections of the fat body away from the cuticle should be carried out after the fixation step.

***Note:*** Due to the fragile nature of fat body, it is recommended that image acquisition occurs on the same day the fat body is mounted. For staining neutral lipids with LipidTOX™ (LipidTOX™ staining is used for lipid droplet analysis), imaging is recommended straight after mounting.

***Note:*** We recommend staining endogenous fluorescently tagged proteins along with DAPI and phalloidin, to help choose Z-stack slices later on for analysis. A list of some endogenous fluorescently tagged ECM proteins that work well in muscle and fat body has been provided in Table 1.

**Figure 5:**
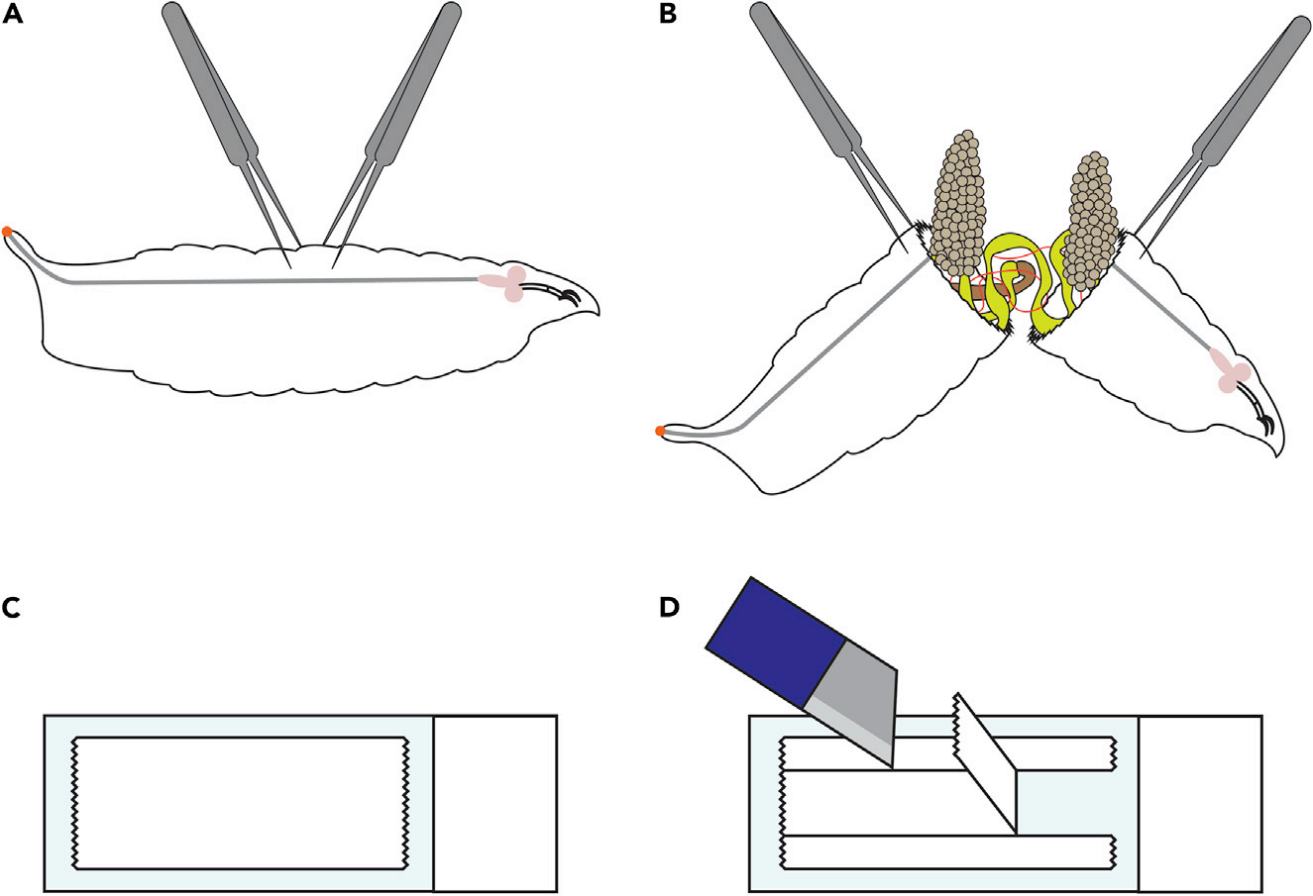
Diagrams of *Drosophila* fat body dissections and slide preparation for fat body mounting (A and B) Diagram of *Drosophila* fat body dissections. (A) Two pairs of forceps are used to grip the larva. (B) The larva is pulled in half, exposing the fat body. (C and D) Preparation of a microscope slide for fat body mounting. (C) A piece of tape is placed on the slide. (D) A scalpel is used to cut the tape as indicated, and the middle portion is removed.
44. Pull enough to expose the fat body as much as possible.
45. Repeat steps 43 and 44 for the other larvae in the same well.
46. Leave the larvae in the fixative for 40 min at RT, without agitation.

***Note:*** See “troubleshooting: problem 3”

41. Transfer larvae from the housing vial to a 6-place porcelain spotting plate with cold 1× PBS (this stops larvae from moving and makes dissections easier). You can have multiple larvae per well of the dish, we recommended between three to five larvae.
42. Under the microscope, change the cold PBS to 4% formaldehyde. Use around 200 μL, as this should be enough to fully submerge the larvae.
43. Using two pairs of forceps, grip the cuticle of one of the larvae as shown in Figure 5A and pull the larva as shown in Figure 5B.

47. After fixation:
  a. For endogenous fluorescently tagged proteins, replace the fix with 200 μL of 0.2% PBST and use this for subsequent washes and stains.
    i. Wash twice in PBST, 10 min per wash.
    ii. During this time, remove all non-fat body tissues from the wells.
  b. For LipidTOX™ staining used in the lipid droplet analysis, add 200 μL of PBS (note: do not use PBST, as Triton affects the integrity of the lipid droplets), and use this for subsequent washes and stains.
    i. Wash twice in PBS, 10 min per wash.
    ii. During this time, remove all non-fat body tissues from the wells.

**CRITICAL:** Take care not to touch the fat body, as these tissues are delicate in nature.

**Table 1:** Fly strains with endogenous fluorescently tagged ECM proteins suitable for use in muscle and fat body

Fly strains	Muscle visualization	Fat body visualization
Viking-GFP	Assembles along the membrane of muscle segments	Forms plaques along cell membrane
Trol-GFP	Assembles along the membrane of muscle segments	Forms plaques along cell membrane
Rhea-mCherry	Assembles at muscle attachment sites	Lines the cell membrane
Nidogen-GFP	Lines the membrane of muscle segments	Forms plaques along cell membrane

### Staining of fixed larval fat body

**Timing: 1 h30 min**

Here we describe the staining of fixed fat body with either DAPI and phalloidin or LipidTOX™.

48. For staining:
  a. For endogenous fluorescently tagged proteins, remove the 0.2% PBST, and add DAPI/phalloidin diluted in 200 μL of 0.2% PBST per well of the spotting plate. We use a DAPI concentration of 1:100, and a phalloidin concentration of 1:200.
  b. For LipidTOX™ staining, remove the PBS, and add LipidTOX™ diluted in 200 μL of PBS per well of the spotting plate. We use a concentration of 1:1,000 for LipidTOX™ staining.
49. For either staining, stain the fillets in the dark for one hour at RT without agitation.
50. Once the staining is complete;
  a. For DAPI/phalloidin staining, replace the DAPI/phalloidin mixture with 200 μL 0.2% PBST, and wash three times (10 min each) at RT.
  b. For LipidTOX™, replace the PBS/LipidTOX™ mixture with 200 μL PBS, and wash three times (10 min each) at RT.

### Mounting of stained larval fat body

**Timing: 15 min**

Here we describe the mounting of stained fat body for imaging.

51. Once ready to mount;
  a. For endogenous fluorescently tagged proteins stained with DAPI/phalloidin, remove the 0.2% PBST, and replace with 80% glycerol.
    ***Note:*** This will make the fat body very difficult to see, and it is recommended to do the next step under a microscope.
  b. For LipidTOX™, we recommend mounting directly in PBS, as glycerol can change the morphology of the lipid droplets.
52. Cut off the tip of a P1000 pipette tip to increase the size of the opening. This allows the fat body to be transferred without it getting caught or squeezed by the tip entrance.
53. Take a small piece of tape and place it on a glass slide as shown in Figure 5C.
54. Using the scalpel/Stanley knife, cut an open strip through the center of the tape as shown in Figure 5D, to create a spacer. This is where we will mount the fat body. The use of the tape prevents the coverslip from squashing the fat body and thus damaging its integrity.
55. Using the cut P1000 pipette tip and a P1000 pipette;
  a. For endogenous fluorescently tagged proteins stained with DAPI/phalloidin, transfer the glycerol containing the fat body to the space created within the tape on the slide. Arrange the fat body, so that the pieces of fat body lie flat.
  b. For LipidTOX™ samples, transfer the PBS containing the fat body to the space created within the tape on the slide. Arrange the fat body, so that the pieces of fat body lie flat.

**CRITICAL:** The LipidTOX™ stained fat body is suspended in PBS, and can sometimes stick to the walls of the pipette tip. Be gentle when transferring these fat bodies, and pipette plenty of PBS while you do so to avoid the fat body sticking to the walls.

56. Using a P200 pipette, remove excess glycerol/PBS from the slide.
57. Gently place a coverslip on top, and seal the edges of the coverslip with nail varnish.

### Imaging of stained larval fat body

**Timing: Varies based on confocal**

Here we describe the settings required for imaging the fat body so that they can be analyzed in the sections below. We have not provided step-by-step methods for the imaging itself, as this differs depending on the microscope used.

***Note:*** Fat body staining and/or fluorescently tagged protein intensity can be quite variable between different individuals of the same experimental condition, and even between the different fat body tissues taken within one individual. To help reduce variability, we take the same piece of fat body for each individual; the long, continuous pieces of lateral fat body (most larvae have two). We also ensure to only use samples where the fat cells are not detached/detaching from one another. In addition, the use of DAPI helps to choose fat cells from the same layer within a sample, as well as can provide guidance of choosing layers of a similar depth into the tissue in other samples.

***Note:*** Fat bodies were imaged on an Olympus FV3000 confocal microscope using a 40×/0.95 NA UPLXAPO air objective.

### Settings required for acquisition

The following settings are utilized to acquire the images utilized for the FIJI analyses.

58. Acquire a Z-stack to ensure the most representative selection of lipid droplets/ECM plaques in the fat body can be measured. As the aim is not to image the precise volume of the fat body, an interval of 1 or 2 μm was used to speed up Z-stack acquisition and reduce file size.
  a. As the lipid droplets and ECM components can be very small, at least a 40× objective is recommended for acquisition as this will allow you to resolve smaller droplets, ECM plaques, and the cell walls.
  b. A higher image size of 1024 × 1024 pixels is recommended as resolution needs to be high to sufficiently resolve smaller droplets and ECM components.
  c. Kalman averaging of 2 or higher is recommended to remove noise, increase signal to noise ratio, and help resolve smaller lipid droplets and ECM components.

***Note:*** See “troubleshooting: problem 4”

### Quantification of lipid droplets from imaged larval fat body

**Timing: 2 min per fat body**

Here we describe the procedure for analyzing lipid droplet size in larval fat body.

***Note:*** The macro has the potential to analyze fat body that has been stained with multiple channels.

**Figure 6:**
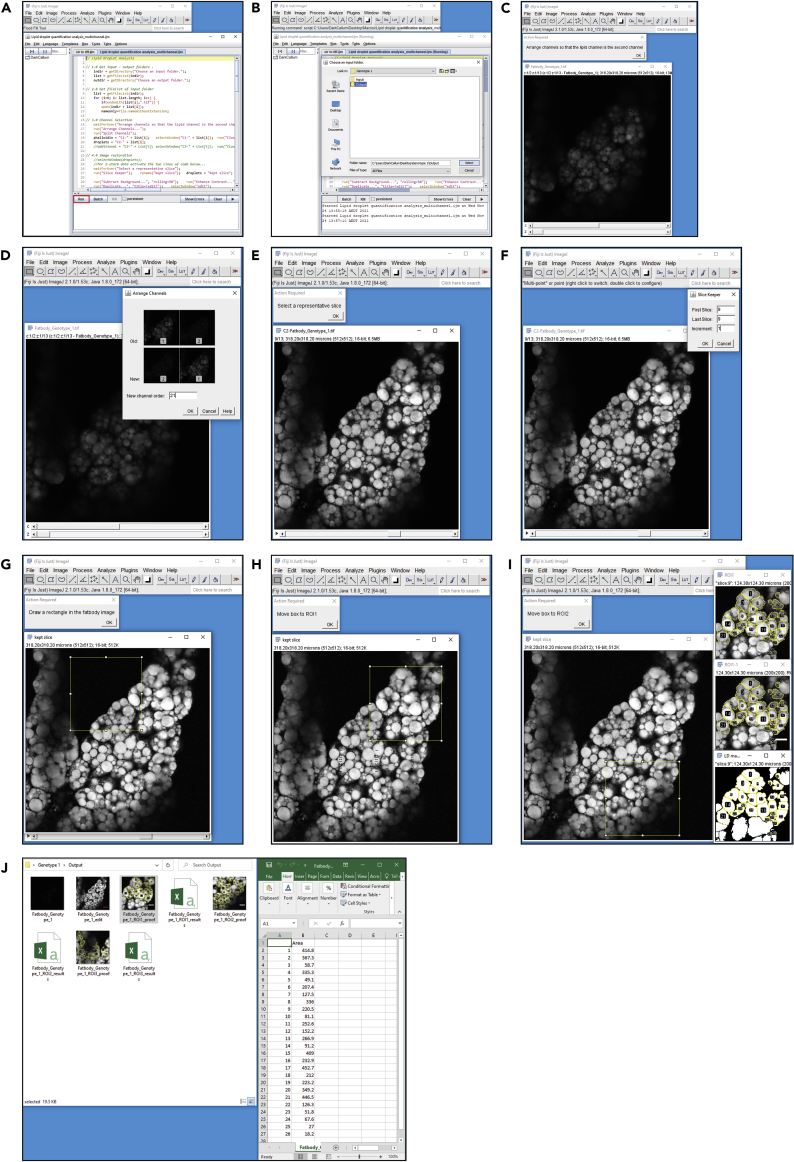
Walkthrough of the lipid droplet quantification macro (A–J) Brief illustrated instructions for the lipid droplet FIJI macro. Detailed instructions can be found in the main text.

***Note:*** The “Lipid droplet quantification analysis_singlechannel” and “Lipid droplet quantification analysis_multichannel” macros are included as uploaded files associated with this protocol (Data S3 and S4 respectively).

**Figure 7:**
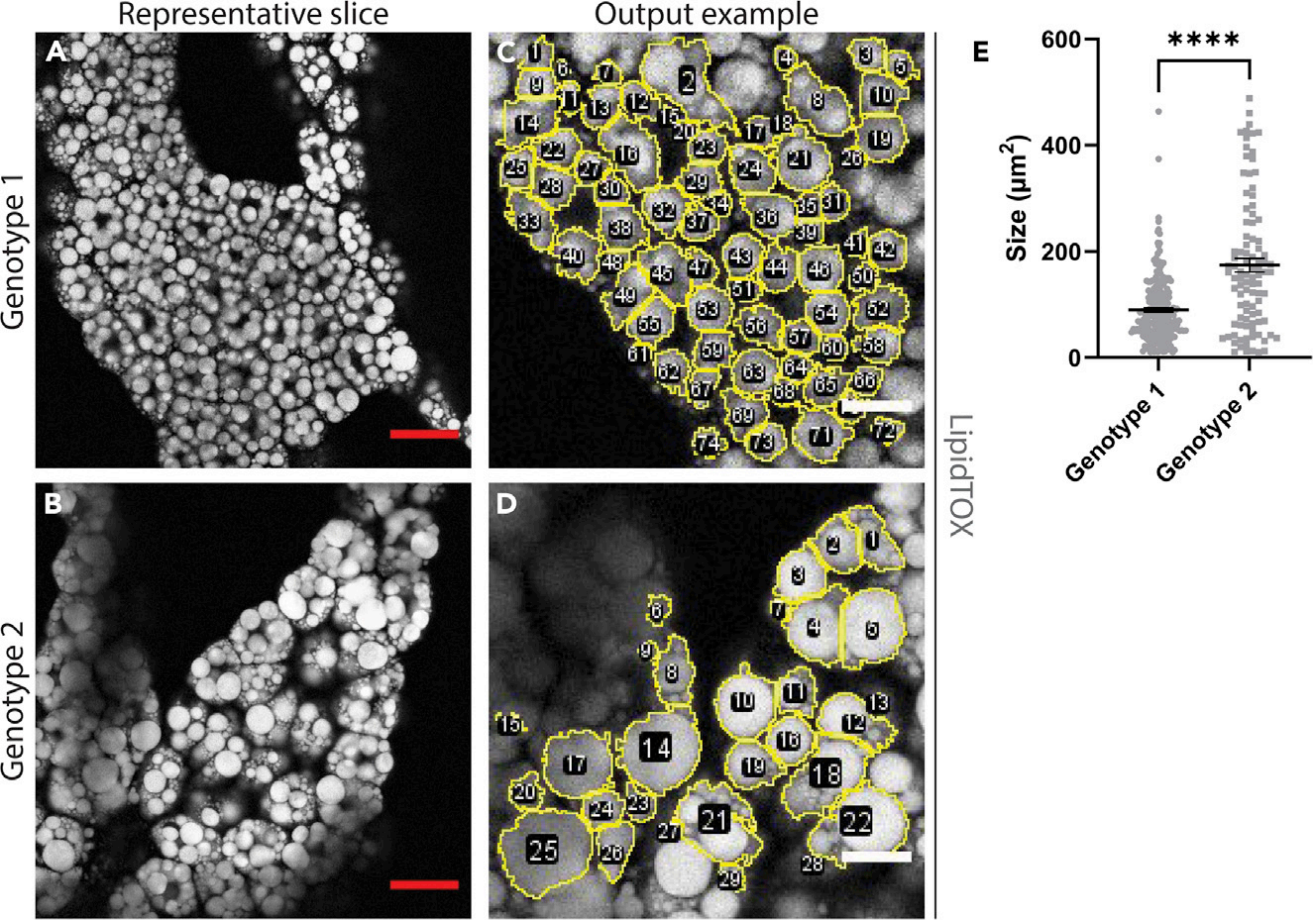
An example of the use of the FIJI lipid droplet macro (A and B) Representative slices from Z-stacks of fat body from two different genotypes. Scale bars are inserted by the FIJI macro, = 50 μm. (C and D) Examples of ROI outputs from (A and B). Scale bars are inserted by the FIJI macro, = 20 μm. (E) Quantification of the lipid droplets found in (C and D). Error bars = ± SEM, ∗∗∗∗ = *p* < 0.0001.
67. You will then be asked to draw a rectangle or square and click ‘OK’ (Figure 6G). Regardless of the size square you draw, it will automatically make a 200 μm × 200 μm square.

***Note:*** This does not need to be around the area you are looking for yet, it is simply to draw an ROI with the correct size, which will be used to select three ROIs you will place in the next steps. If you click ‘OK’ without setting a rectangle or square, it will generate one for you.

59. Separate images into folders according to the experimental conditions used, for example, genotype, drug treatment, etc.
60. Within the respective folders, create an ‘Input’ and an ‘Output’ folder. Move the images into the ‘Input’ folder for each respective group. See Figures 3A–3E for an example of the “oir to tiff” macro used in steps 61 and 62.
61. Import the “oir to tiff” macro into FIJI (Data S1), and hit ‘Run’. Macros can be imported by dragging and dropping the macro file into FIJI.
62. Select the ‘Input’ folder for your first group, and the ‘Output’ folder for where the macro will deposit the converted files. It will run through the images in the ‘Input’ folder, and output them as converted tiff files in the ‘Output’ folder.
63. Once the command has finished, Import the “Lipid droplet quantification analysis_singlechannel” or “Lipid droplet quantification analysis_multichannel” (either channel option depending on your needs) macro into FIJI, and hit ‘Run’ (Figure 6A).

64. Select the ‘Output’ folder from where you sent the converted files from step 62 (Figure 6B).
65. The command will begin, and will show an image of the first fat body.
  a. If you are using the multi-channel macro, it will ask you to organize the channels to select the lipid droplet channel. Follow the instructions accordingly (Figures 6C and 6D).
  b. If you are using the single channel macro, it will skip to the next step.
66. You will be asked to select your representative slice (Figure 6E). Scroll to find a slice that is around 4–5 slices from the top of the stack, one that is bright and shows the diameter of the lipid droplets, and make sure there is a defined separation between lipid droplets (See Figures 7A and 7B).
  a. Click ‘OK’. A pop-up box will ask for the slices you are looking to keep. Enter the number of the slice you have kept in both the ‘First Slice’ and ‘Last Slice’ boxes, and set the increment to ‘1’ (Figure 6F).

68. Move the ROI to the first position you want to analyze and click ‘OK’ (Figure 6H).

***Note:*** If you click ‘OK’ before moving the ROI to new position for the second or third ROIs, the macro will encounter an error and will need to be restarted.

69. Repeat step 68 for the second and third ROIs (Figure 6I).
70. Proceed through each fat body in the given folder. Each fat body will give a .csv output that contains the number of lipid droplets detected, and their size. See Figure 6J for an example of the outputs from one fat body, and the .csv output of one ROI.

***Note:*** Pay attention to the output images that show what the macro has chosen as a lipid droplet. It will have assigned numerical values to each lipid droplet, so if it has incorrectly detected a lipid droplet you can select its value and remove it if need be.

71. Repeat steps 61–70 for the other experimental groups.

### Quantification of ECM components from imaged larval fat body

**Timing: 3 min per fat body**

Here we describe the procedure for analyzing ECM components using FIJI.

***Note:*** It is recommended that you only use one channel for the ECM protein of interest. The experimental and control fat bodies should always be acquired in parallel, and the same confocal setting should be applied to both. Acquire the brightest sample first and apply this setting to the dimmer samples.

**CRITICAL:** It is very important to acquire these images as soon as possible after mounting.

72. Import Z-stack of fat body with a fluorescently tagged ECM protein.
73. Isolate the slice that is 4^th^ or 5^th^ from the top of the stack, which also has the brightest DAPI signal. Make sure to only measure the slice that contains the ECM signal, for example in Figures 8A, 8B, and 8H.

**Figure 8:**
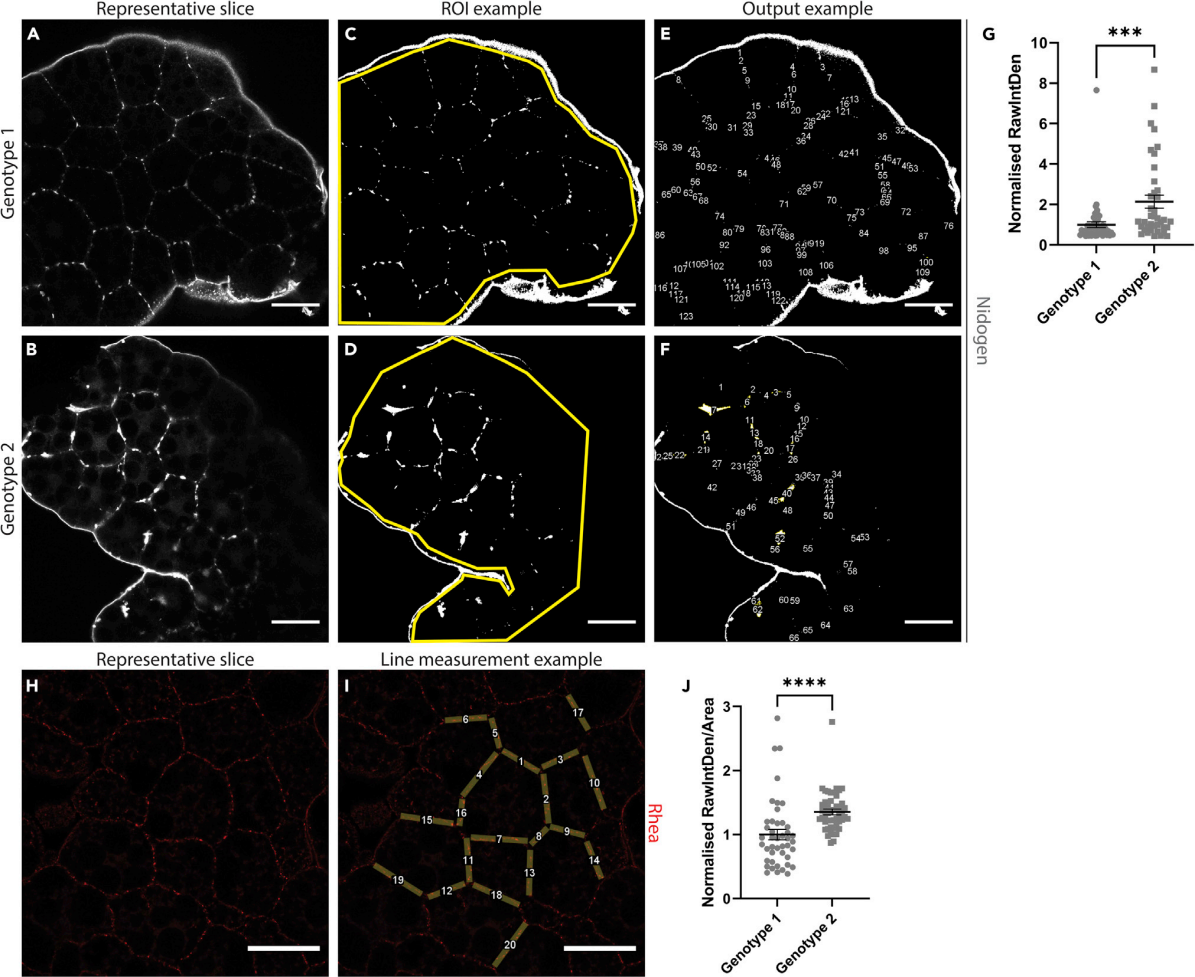
Examples of ECM protein quantifications in *Drosophila* fat body (A and B) Representative slices from Z-stacks of fat body from two different genotypes showing the ECM protein Nidogen assembling as plaques. (C and D) Examples of how to draw the ROI on the inner side of the outer fat body edge. (E and F) Particle analysis performed on (C and D). (G) Quantification of the ECM plaques found in (E and F). Error bars = ± SEM, ∗∗∗ = *p* < 0.001. (H) Representative slice from Z-stack of fat body showing the ECM protein Rhea aligning along the cell edge. (I) Example of line measurement for (H). (J) Quantification example of ECM line measurements from (I). Raw Integrated Density is divided by the area of the line measurement, and values are normalized to the average of the control values. Error bars = ± SEM, ∗∗∗∗ = *p* < 0.0001. All scale bars = 50 μm.
74. Use the following instructions based on what formation the ECM component of interest follows:
75. For ECM components that assemble as ‘plaques’ (See Figures 8A–8G), in order to measure the size of the plaques: apply the “Auto Threshold” feature (Image>Adjust>Auto Threshold),
  a. Choose “Try all” to first assess which setting is the most reflective of the image.
  b. Then apply the most precise thresholding algorithm under “Auto Threshold” to your image.
    **CRITICAL:** It is important to use the same setting for all experimental conditions.
    ***Note:*** See “troubleshooting: problem 5”.
  c. Usually there is some signal around the outer edges of the fat body. You do not want this to be measured as part of the analysis, so you need to select only the internal regions of the fat body. Using the “Polygon Selections” tool, draw an ROI around the internal region of the fat body, excluding any outer edge signals. See Figures 8C and 8D for examples.
  d. Utilize the “Analyze Particles” tool (Analyze>Analyze Particles) to count the number of ECM ‘plaques’.
    i. Make sure “Display Results”, “Add to Manager”, and “Exclude on edges” are ticked.
    ii. Set the values in the “Size” box to “1.00-infinity”. This helps to ensure that background singular pixels are not measured.
    iii. Set the values for the “Circularity” box to “0.00–1.00”.
    iv. You will also need “Integrated density” ticked in “Set Measurements” (Analyze>Set Measurements).
    v. It will output the results in a separate window.
  e. Measure using “RawIntDen”.
76. For ECM components that assemble along the cell edge (See Figures 8H–8J), draw a line of 10 μm width along the membrane (Figure 8I), where to draw this line can be guided by the phalloidin staining that you have acquired (Double click on the “Line segment” tool to set line width). Collect around 10–20 line segments per image.
  a. It is suggested to use the “ROI Manager” tool (Analyze>Tools>ROI Manager) to help keep track of which line segments have been measured. You will also need “Area” and “Integrated density” ticked in “Set Measurements” (Analyze>Set Measurements).
  b. Once you have collected all the line segments for one image, select all the ROIs and click “Measure” in the ROI Manager.
  c. Calculate the fluorescent intensity by dividing “RawIntDen” by the area of the line measurement.
  d. Normalize each value to the average of the control values.

## Expected outcomes

The protocols included here describe how to analyze cachectic phenotypes in the *Drosophila* larvae. In the context of muscle, this protocol demonstrates how larvae can be quickly screened to examine the degree of which the experimental condition affects muscle detachment. Examples of intact and detached fillets can be seen in Figures 4C and 4D respectively. The scores from these fillets can be easily quantified and analyzed with statistical methods, as seen in Figure 4E. We would expect that cachectic muscles show a decrease in percentage area when compared to wildtype muscle.

In the context of fat body, this protocol demonstrates how to quantify lipid droplet size in a systematic and semi-automated fashion. Examples of measured lipid droplets can be seen in Figures 7C and 7D. We expect cachectic fat body to show larger lipid droplet size when compared to wildtype fat body. Due to the large number of small lipid droplets regardless of genotype, it is likely that the data points will be heavily skewed towards the lower values (see Figure 7E). However, large shifts in the mean created by the larger droplets can demonstrate changes in lipid droplet size. In addition, this protocol shows how to quantify the accumulation of ECM proteins in the fat body. In particular for ECM plaques, rather than purely measuring fluorescence levels, this protocol demonstrates how to measure changes in plaque size, which is not always correlated with total fluorescence levels. Examples of these size differences can be seen in Figures 8C and 8D.

## Quantification and statistical analysis

The outputs of all protocols listed in this paper can be statistically analyzed using simple comparison tests, such as the Student’s T-test, or a One-Way ANOVA, or their nonparametric counterparts. The degree of complexity for your comparisons will depend on your experimental conditions.

All data was plotted using GraphPad Prism version 9.0.2 for Windows, GraphPad Software, San Diego, California USA, www.graphpad.com.

## Limitations

The muscle fillet macro is quite robust, however it isn’t recommended to use it for investigating minor differences, for example, differences in detachment scores less than 5%. Given that detachment scores of wildtype fillets can vary between 80%-90%, the natural variation in even intact fillets means that minor differences may not be real.

The lipid droplet macro is limited in its capacity to detect droplet boundaries when the lipid droplets are merged.

## Troubleshooting

### Problem 1

Mounting of muscle fillets upside down (i.e., cuticle side up). See step 25.

### Potential solution

The muscle fillets are quite robust, therefore, if the fillets are mounted the wrong way round, they can be remounted, by removing the coverslip, and washing/mounting again in the right orientation.

### Problem 2

The muscle fillet macro is detecting more or less than what is reflective by eye. See step 38a.

### Potential solution

The acquired images can be manually adjusted to ensure the phalloidin stained muscles are still picked up by the macro. A manual threshold can be applied at the step where the macro prompts to “check ROI”. You can manually adjust the threshold by using the Tools “Image>Adjust>Threshold” at this step, to ensure the macro is picking up all the phalloidin staining.

### Problem 3

Fat body seems extremely fragile. See step 46.

### Potential solution

The fat body can be fixed for up to an hour, which would enhance the tissue integrity.

### Problem 4

Fat body lipid droplet staining appears blurry or extremely bright. See step 58.

### Potential solution

This sample is probably not going to be comparable to other samples, however, in the future, more washing in PBS would overcome this issue.

### Problem 5

FIJI is unable to complete thresholding for a particular algorithm for all samples when examining ECM plaques, or after thresholding, the ECM plaques are not reflective of what you see by eye for all samples. See step 75b.

### Potential solution

If this occurs, you may need to choose a different algorithm that can be completed on all samples. Repeat step 75, and choose a different thresholding algorithm that is still reflective of the ECM plaques you see by eye in the image. Check with your other samples that this algorithm can be completed on them as well, and make sure to use the same setting for the experiment and control conditions before continuing.

## Resource availability

### Lead contact

Further information and requests for resources and reagents should be directed to and will be fulfilled by the lead contact, Louise Cheng, louise.cheng@petermac.org.

### Materials availability

This study did not generate new unique reagents.

## Data Availability

The published article includes all macros generated during this study.

## Supplemental information

Data S1. Oir to tiff macro, related to steps 33 and 61

Data S2. Fillet muscle detachment analysis macro, related to step 35

Data S3. Lipid droplet quantification analysis_singlechannel macro, related to step 63

Data S4. Lipid droplet quantification analysis_multichannel macro, related to step 63
